# Multiparametric Brain Hemodynamics Imaging Using a Combined Ultrafast Ultrasound and Photoacoustic System

**DOI:** 10.1002/advs.202401467

**Published:** 2024-06-17

**Authors:** Haoyang Chen, Shubham Mirg, Prameth Gaddale, Sumit Agrawal, Menghan Li, Van Nguyen, Tianbao Xu, Qiong Li, Jinyun Liu, Wenyu Tu, Xiao Liu, Patrick J. Drew, Nanyin Zhang, Bruce J. Gluckman, Sri‐Rajasekhar Kothapalli

**Affiliations:** ^1^ Department of Biomedical Engineering The Pennsylvania State University University Park PA 16802 USA; ^2^ Center for Neural Engineering The Pennsylvania State University University Park PA 16802 USA; ^3^ Institute for Computational and Data Sciences The Pennsylvania State University University Park PA 16802 USA; ^4^ Department of Engineering Science and Mechanics The Pennsylvania State University University Park PA 16802 USA; ^5^ Department of Biology The Pennsylvania State University University Park PA 16802 USA; ^6^ Department of Neurosurgery The Pennsylvania State University University Park PA 16802 USA; ^7^ Penn State Cancer Institute The Pennsylvania State University Hershey PA 17033 USA; ^8^ Graduate Program in Acoustics The Pennsylvania State University University Park PA 16802 USA

**Keywords:** Brain hemodynamics, Cerebrovascular reactivity, Functional ultrasound, Multimodal imaging, Photoacoustic imaging

## Abstract

Studying brain‐wide hemodynamic responses to different stimuli at high spatiotemporal resolutions can help gain new insights into the mechanisms of neuro‐ diseases and ‐disorders. Nonetheless, this task is challenging, primarily due to the complexity of neurovascular coupling, which encompasses interdependent hemodynamic parameters including cerebral blood volume (CBV), cerebral blood flow (CBF), and cerebral oxygen saturation (SO_2_). The current brain imaging technologies exhibit inherent limitations in resolution, sensitivity, and imaging depth, restricting their capacity to comprehensively capture the intricacies of cerebral functions. To address this, a multimodal functional ultrasound and photoacoustic (fUSPA) imaging platform is reported, which integrates ultrafast ultrasound and multispectral photoacoustic imaging methods in a compact head‐mountable device, to quantitatively map individual dynamics of CBV, CBF, and SO_2_ as well as contrast agent enhanced brain imaging at high spatiotemporal resolutions. Following systematic characterization, the fUSPA system is applied to study brain‐wide cerebrovascular reactivity (CVR) at single‐vessel resolution via relative changes in CBV, CBF, and SO_2_ in response to hypercapnia stimulation. These results show that cortical veins and arteries exhibit differences in CVR in the stimulated state and consistent anti‐correlation in CBV oscillations during the resting state, demonstrating the multiparametric fUSPA system's unique capabilities in investigating complex mechanisms of brain functions.

## Graphical abstract

In this work, a multimodal functional ultrasound and photoacoustic (fUSPA) imaging system capable of comprehensively mapping brain‐wide hemodynamics (microvascular blood volume, blood flow, oxygen saturation, and vasoreactivity) in response to various stimuli with high spatiotemporal resolutions is presented. The head‐mountable fUSPA device has the potential to gain new insights into the mechanisms of several neurological disorders, including studies on behaving and freely moving animals.

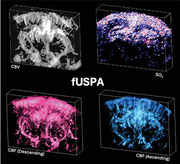

## 1 Introduction

Cerebral hemodynamics, controlled by various physiological factors like autoregulation, neurovascular coupling, and vascular reactivity, is vital for brain health as well as the exchange of gases and nutrients.^[^ ^1^ ^]^ Cerebral blood volume (CBV), cerebral blood flow (CBF), and cerebral oxygen saturation (SO_2_) are three important hemodynamic parameters commonly studied for assessing cognitive function and help gain insights into the diagnosis and treatment of neurological disorders.^[^ ^2^ ^]^ Though CBV, CBF, and SO_2_ are interdependent with significant coupling,^[^ ^3^, ^4^ ^]^ technical difficulties limit the current modalities to individually map these cerebral hemodynamic parameters in the deep brain. For example, functional magnetic resonance imaging (fMRI) based blood oxygen level‐dependent (BOLD) signal is widely used in neuroscience to non‐invasively study whole‐brain hemodynamic changes.^[^ ^5^ ^]^ However, fMRI exhibits low spatial resolution, and the BOLD signal is a mix of several hemodynamic factors,^[^ ^5^, ^6^ ^]^ making interpretation confounding. Moreover, the noise and the bulky size of fMRI instrumentation restrict its applicability in studies involving naturally behaving animals.^[^ ^6^ ^]^ On the other hand, optical imaging methods, such as two‐photon fluorescence microscopy, intrinsic optical signal imaging, and laser speckle contrast imaging^[^ ^7^ ^]^ can map changes in CBF, CBV, and individual vessel diameter in the rodent brain with high resolution. Yet the imaging depth is limited to superficial regions (<2 mm inside the brain) and often requires surgical procedures to create optically transparent windows.^[^ ^8^ ^]^

Towards this, ultrasound imaging was considered a promising alternative due to its high spatiotemporal resolutions compared to fMRI as well as better penetration depth than optical imaging. Recently, ultrafast ultrasound (UFUS) based on plane wave imaging has emerged as a promising technique, offering multiparametric (CBV and CBF) microvasculature information from moving scatterers within blood vessels, such as red blood cells or injected microbubbles (MBs). Power Doppler ultrasound imaging (also known as functional ultrasound—fUS) uses ultrafast multiangle coherent plane‐wave compounding to map CBV changes from moving red blood cells with high spatial (100 µm × 100 µm × 300 µm voxel) and temporal (300 msec–1 sec) resolutions.^[^ ^9^, ^10^ ^]^ These CBV changes have also shown good agreement with direct measures of hemodynamics at the single vessel level using 2‐photon microscopy.^[^ ^11^ ^]^ Meanwhile, quantitative CBF change maps can also be obtained by fitting autocorrelation functions to the acquired ultrasound speckles.^[^ ^12^ ^]^ By intravenously injecting acoustic contrast agents MBs and applying MB localization and tracking methodologies, UFUS further provides super‐resolved ultrasound localization microscopy (ULM) images of vascular anatomy, vertical blood flow direction, and flow speed with a spatial resolution that is beyond the ultrasound wave diffraction limit.^[^ ^13^ ^]^

While UFUS has shown promise in CBV and CBF mapping for deep brain imaging, functional SO_2_ information remains inaccessible. In this regard, multi‐spectral photoacoustic (MSPA), a molecular imaging technique, can be employed. MSPA is a hybrid imaging modality, where optical pulses of different wavelengths illuminating the tissue are converted to broadband ultrasound waves by light‐absorbing molecules such as hemoglobin in red blood cells, and ultrasound detectors are used to capture these photoacoustic waves. Based on the differential optical absorption of oxy‐hemoglobin (HbO) and deoxy‐hemoglobin (HbD), MSPA can map time‐ and depth‐ resolved total hemoglobin concentration and SO_2_ changes at ultrasound resolutions.^[^ ^14^ ^]^ In addition to this label‐free vascular information, MSPA can provide a wide range of exogenous molecular contrast via the administration of optical contrast agents such as indocyanine green (ICG)^[^ ^15^, ^16^ ^]^ or genetically encoded light‐absorbing proteins.^[^ ^15^, ^17^ ^]^

Although UFUS and MSPA have individually demonstrated the promise for brain imaging, their isolated use and lack of co‐registration of interdependent hemodynamic parameters limits the scope for studying complex brain functions. Recent studies have reported combined ultrasound and photoacoustic technologies for high‐resolution functional brain imaging.^[^ ^18^, ^19^ ^]^ Yet, no research has effectively integrated the MSPA and UFUS imaging into a single platform for comprehensive hemodynamic analysis of the deep brain in response to stimuli. Here, we report a multimodal functional ultrasound and photoacoustic (fUSPA) imaging system with interleaved UFUS and MSPA acquisitions, capable of mapping multi‐parametric (CBV, CBF, SO_2_) hemodynamics alongside exogenous molecular imaging of the brain, using a compact head‐mountable device. In the following sections, we first present various characterization results of the fUSPA imaging system and its imaging capabilities using phantom, ex vivo, and in vivo rat brain studies. Then we show the feasibility of optical and ultrasound contrast agent enhanced in vivo brain imaging. Next, using hypercapnia stimulation‐induced in vivo rat models, we study brain‐wide cerebrovascular reactivity (CVR) differences at single‐vessel resolution using dissected high‐resolution and high‐sensitivity hemodynamic information. Furthermore, fUSPA was applied to study the resting‐state brain activity of rats, which revealed distinct venous dynamics compared to the cortical dynamics. In the future, the high‐resolution, high‐sensitivity, deep brain, multiparametric hemodynamic mapping enabled by fUSPA may help gain insights into brain functions in response to various stimuli, mental illnesses,^[^ ^20^ ^]^ brain cancer,^[^ ^21^ ^]^ and transient and complex neurological disorders such as epilepsy.^[^ ^22^ ^]^

## 2 Results

### 2.1 Functional Ultrasound and Photoacoustic (fUSPA) Imaging System

**Figure 1:**
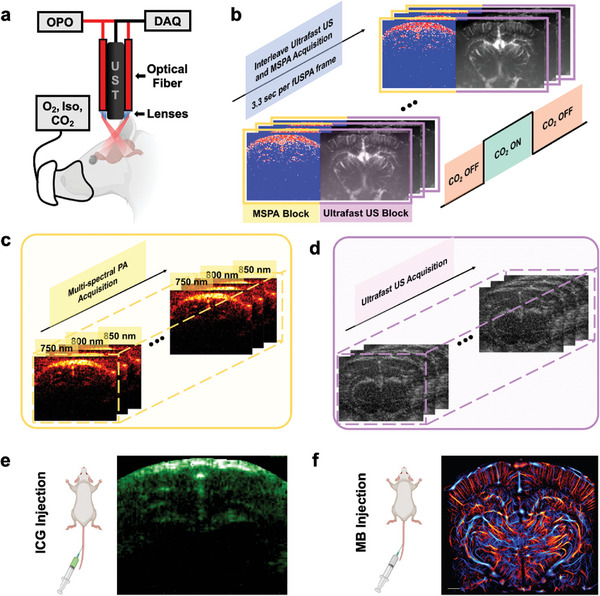
The schematic of the multimodal functional ultrasound and photoacoustic (fUSPA) imaging system and the interleaved imaging sequences. a) The overall schematic of the multimodal fUSPA imaging system using a compact head‐mountable imaging device that integrates ultrasound transducer (UST) and optical fiber bundles. The UST probe is connected to the Verasonics data acquisition system (DAQ) to transmit and receive acoustic signals. The fiber optic bundle is connected to the tunable optical parametric oscillator (OPO) laser and delivers light pulses into the brain. The imaging device is water coupled to the transparent chronic cranial window on the rat head. b) The interleaved multimodal fUSPA data acquisition. Each fUSPA frame consists of one multispectral photoacoustic (MSPA) data block and one ultrafast ultrasound (UFUS) data block. The frame rate of fUSPA is ≈3.3 frames per second. c) Each MSPA data block consisted of three photoacoustic images acquired at three wavelengths—750, 800, and 850 nm—in a sequence at 100 ms per PA frame. d) In each UFUS data block, 150 plane‐wave angle compounded images were acquired to generate one power Doppler‐based cerebral blood volume (CBV) image and autocorrelation velocimetry‐based cerebral blood flow (CBF) map. e) MSPA imaging of intravenously injected photoacoustic contrast agent indocyanine green (ICG) generates vascular perfusion maps. f) UFUS imaging of intravenously injected ultrasound contrast agents, microbubbles (MBs), provides a super‐resolved microvasculature imaging using ultrasound localization microscopy principles.

We developed a multimodal fUSPA imaging system that can perform interleaved UFUS and MSPA imaging of the brain using a compact imaging head. The device tightly integrated a 15 MHz high‐frequency ultrasonic transducer (UST) and fiber optic light delivery through lenses such that light and ultrasound fields co‐align 7 mm away from the transducer surface (**Figure** **1**a, Experimental Section; Figure S1, and Section S1, Supporting Information). The optical fiber bundle was coupled to a wavelength‐tunable nanosecond pulsed optical parametric oscillator (OPO) laser and the UST was connected to a research ultrasound data acquisition system (DAQ)—Vantage 256 for performing acoustic transmit–receive beamforming and synchronizing triggers with the laser. The Vantage was sequence programmed to continuously obtain fUSPA frames (each consisted of one MSPA and one UFUS block) at 3.3 s per frame (Figure 1b, Figure S2, Supporting Information and Experimental Section) to provide the following multiparametric functional cerebral hemodynamic maps: cerebral SO_2_ map derived from the spectral unmixing of three‐wavelength MSPA data (Figure 1c, Experimental Section) and CBV and CBF maps obtained from UFUS based multi‐plane wave angle compounded data (Figure 1d, Figure S2, Supporting Information, Experimental Section). Additionally, the fUSPA platform can be expanded to facilitate exogenous contrast‐enhanced brain imaging using a variety of optical and ultrasound contrast agents and respective molecular probes, including MSPA‐based exogenous molecular imaging (Figure 1e) and ULM‐based super‐resolution vasculature imaging (Figure 1f, Experimental Section) by intravenous injection of respective optical (e.g., ICG) and ultrasound (e.g., MBs) contrast agents. The integrated imaging of anatomical, functional, and molecular information of the brain at scalable depths and spatial resolutions is a distinct advantage of our fUSPA system, setting it apart from other modalities available.

### 2.2 Multiparametric Hemodynamic Imaging of the Brain

Prior to in vivo imaging, multiple characterization steps were carried out to study the performance of the fUSPA imaging system. This includes pulse‐echo and PA A‐line characterization using a flat metal target, resolution analysis using a wire target phantom, and functional and molecular imaging validation using phantoms consisting of blood flow, SO_2_, and ICG content (Section S2, Figures S3 and S4F, Supporting Information).

**Figure 2:**
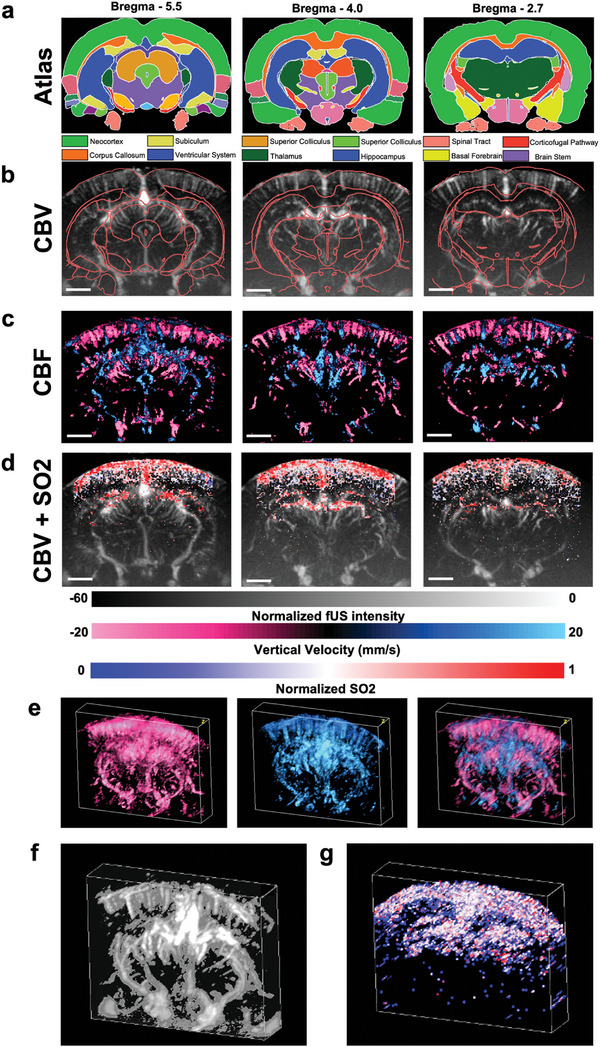
In vivo ultrafast ultrasound (UFUS) and multispectral photoacoustic (MSPA) imaging of rat brains provided dissected multiparametric hemodynamic brain activity maps. a) The coronal rat brain atlas maps at Bregma –5.5, Bregma −4.0, and Bregma –2.7 locations^[^ ^44^ ^]^ with different brain regions in different color codes. b) Three CBV images and c) three CBF images were generated from the UFUS data acquired from the three Bregma locations and overlaid with the respective atlas contours in red color. In the CBF map, the vertical direction of the measured flow is color‐coded, where red color denotes descending flow (downward) and blue color denotes ascending flow (upward). d) The MSPA data‐based unmixed oxygen saturation (SO_2_) maps at the three Bregma locations are co‐registered onto the grayscale CBV images. Volumetric maps of e) CBF f) CBV and g) SO_2_ were generated from the B‐mode UFUS and MSPA data acquired with linear scanning of the imaging device. In e) red and blue colors denote descending and ascending flow maps and the combined CBF map on the right. CBF: cerebral blood flow; CBV: cerebral blood volume. Scale bars represent 2 mm.

We then performed in vivo interleaved UFUS and MSPA imaging of an anesthetized rat brain by coupling the compact fUSPA imaging head to the chronic cranial window that is transparent to both light and ultrasound (see Experimental Section). (**Figure** **2**) shows multiparametric CBV, CBF, and SO_2_ maps from three distinct coronal locations (Bregma −5.5, −4.0, and −2.7) of the rat brain overlaid with corresponding atlases.^[^ ^23^ ^]^ UFUS data was processed to display both grayscale power Doppler intensity maps representing CBV changes (Figure 2b, Experimental Section) and the color‐coded ultrasound velocimetry maps quantifying CBF (Figure 2c, Experimental Section), with red and blue colors denoting descending and ascending blood flow directions in the microvasculature. The SO_2_ maps for the three coronal brain locations (Figure 2d, Experimental Section) are obtained from the linear spectral unmixing of MSPA frames at wavelengths of 750, 800, and 850 nm (Figure S6, Supporting Information) overlaid with the corresponding CBV maps. Unlike ultrasound‐based CBV and CBF maps, the SO_2_ information was limited to ≈6 mm depth, primarily due to limited light illumination through the cranial window and optical attenuation within the brain tissue. This limitation is consistent with other in vivo B‐mode PA brain imaging studies^[^ ^18^ ^]^ as well as our ex vivo studies (Figure S7, Supporting Information). Furthermore, the significantly higher SO_2_ values obtained in vivo (Figure 2d) compared to ex vivo imaging (Figure S7, Supporting Information) validated the capability of MSPA for monitoring cerebral SO_2_ changes. Additionally, as shown in Figure 2e–g, it is possible to generate volumetric (3D) maps of CBF, CBV, and SO_2_ by combining respective 2D (B‐mode) cross‐sectional images obtained from translating the compact fUSPA imaging head along the anterior‐posterior axis (300 µm step size). These results demonstrated that the fUSPA imaging system can provide brain‐wide multiparametric hemodynamic information in vivo with high spatial resolution and sensitivity.

### 2.3 Exogenous Contrast‐Enhanced Brain Imaging

**Figure 3:**
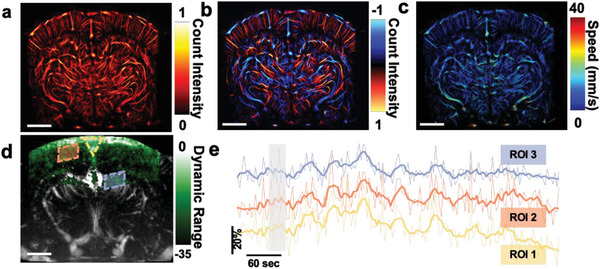
Exogenous contrast agent enhanced fUSPA imaging of rat brain at around Bregma −4.5 location. (a–c) Super‐resolution ultrasound localization microscopy (ULM) imaging of rat brain microvasculature using intravenous injection of microbubbles (MBs). ULM images based on a) MB count intensity and b) MB count intensity color‐coded with vertical flow direction; red and blue colors denote the bubbles moving downward and upward, respectively. c) By calculating the displacement and corresponding frame rate, an averaged blood speed map is generated. d) MSPA imaging‐based vascular perfusion map (green color) using intravenously injected indocyanine green (ICG) co‐registered with UFUS imaging based gray colored CBV map. Three ROIs with three different colors are marked in different brain regions. e) The plots of ICG time activity averaged over the three ROIs showed the relative ICG changes during pre‐injection, injection, and post‐injection periods. The gray shaded area represents the time during ICG injection. The bolded lines represent the smoothed average ICG changes in the ROI. MSPA: multispectral photoacoustic; CBV: cerebral blood volume; ROI: region‐of‐interest. The scale bar of the images represents 2 mm.

We studied the exogenous contrast‐enhanced brain imaging capabilities of the fUSPA system by intravenous injections of US and PA contrast agents in conjugation with UFUS and MSPA imaging, respectively. By localizing and tracking the intravenously injected size‐isolated MBs (peak diameter: 4–5 µm, concentration: 10^8^ MBs mL^−1^. See Experimental Section for details) across multiple compounded frames acquired during the 10‐min UFUS imaging period, we generated a super‐resolved (10 µm × 10 µm) ULM images of brain vasculature covering the whole cross–section of the rat brain (**Figure** **3**a).^[^ ^24^, ^25^ ^]^ The tracking of microbubbles also allowed us to determine the vertical direction of flow (Figure 3b) and calculate the mean flow speed (Figure 3c) (Experimental Section).

Next, we studied exogenous photoacoustic molecular imaging capabilities of the fUSPA system by intravenously injecting ICG molecules (concentration: 129 µm in sterilized saline), while continuously acquiring MSPA data at 750, 800, and 850 nm wavelengths for a total time period of 10 min (see Experimental Section for details). Linear spectral unmixing of each set of the three‐wavelength MSPA frames provided a map of ICG molecular distribution inside the brain (Figure 3d). The comparison of the three‐wavelength MSPA frame sets acquired at pre‐ and post‐ICG injection shows that the overall brain PA intensity post‐injection increased by 2, 5, and 3 dB for 750, 800, and 850 nm wavelengths, respectively (Figure S8, Supporting Information). The strongest difference observed at 800 nm corresponded well with the peak optical absorbance of ICG in plasma at 800 nm wavelength.^[^ ^26^ ^]^ Additionally, unmixing of all the acquired temporal MSPA frames provided time‐stamped unmixed ICG images, which were used to track the temporal dynamics of ICG molecule accumulation and its subsequent clearance during the post‐injection phase (Figure 3e, Experimental Section). All three region‐of‐interests (ROIs) inside the brain—sagittal sinus (ROI 1), cortical vessels (ROI 2), and hippocampal vessels at ≈3.5 mm depth (ROI 3)—showed peak uptake of ICG ≈2 min post‐injection, which aligned well with the 1.5–3 min ICG half‐life in rats.^[^ ^26^ ^]^

### 2.4 Differential Hemodynamic Responses and Cerebrovascular Reactivity (CVR) Observed Between Singular Arteries and Veins Under Hypercapnia Stimulation

CVR is a measure of vessels’ ability to dilate in response to specific stimuli in order to meet the increased demand for blood supply and is often found altered in diseased or aged cerebral vasculatures compared to healthy counterparts.^[^ ^27^, ^28^ ^]^ In this study, a widely adopted hypercapnia stimulation strategy, the inhalation of 5% carbon dioxide (CO_2_) for a short time, to assess the CVR in various diseases^[^ ^29^ ^]^ was used. This stimulation paradigm was chosen as it elicits global vasodilation as well as elevates oxygen saturation levels inside the brain and other organs.^[^ ^30^, ^31^ ^]^

**Figure 4:**
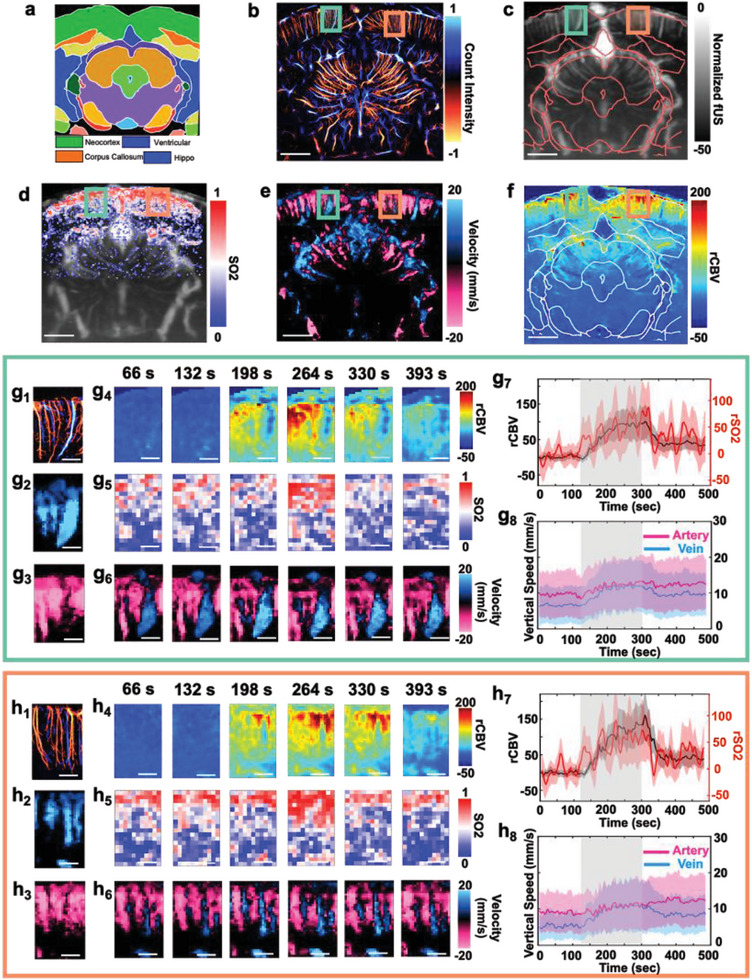
Multiparametric fUSPA imaging revealed regional differences in cerebral vascular reactivity during hypercapnia (CO_2_) stimulation. a) A coronal section map at Bregma −5.8 location of the rat brain atlas^[^ ^23^ ^]^ and b) ULM‐based super‐resolved microvasculature imaging at the Bregma −5.8 location. Red and blue colors denote downward and upward flows. The color bar represents the normalized count intensity. c) The UFUS‐based CBV image overlaid with the atlas from (a), and d) the SO_2_ map obtained from MSPA imaging overlaid onto the corresponding CBV map. e) The CBF map was generated from the velocity autocorrelation of UFUS frames, where red and blue denote downward and upward blood flows. In the cortex region, the descending vessels were arterioles and the ascending vessels were venules. f) The relative CBV (rCBV) change map during hypercapnia (*t* = 260 s) stimulation. Two ROIs from the brain cortex regions in the images Figure 4b–f were enlarged and shown in Figure 4g,h to study regional differences in the hemodynamic responses. In Figure 4g,h, the first subfigures (Figure 4g_1_,h_1_) display ULM based microvascular map inside the corresponding ROI, with a scale bar of 0.5 mm. The second (Figure 4g_2_,h_2_) and third (Figure 4g_3_,h_3_) subfigures display the vessels with ascending and descending flows obtained from the CBF maps of the two ROIs, respectively. Subfigures display (Figure 4g_4_,h_4_) the rCBV, (Figure 4g_5_,h_5_) the SO_2_, and (Figure 4g_6_,h_6_) the CBF dynamics inside the two ROIs for selected time points during the hypercapnia stimulation. The (Figure 4g_7_,h_7_) subfigures plot the averaged rCBV (black line) as well as the averaged rSO_2_ (red line) changes in the respective ROIs during the hypercapnia stimulation. The black and red shaded areas represent the mean +/− standard deviation. The Figure 4g_8_,h_8_ subfigures display differential vertical CBF between the arteries and veins, determined from the flow direction, during the hypercapnia stimulation. The blue and red shaded areas represent the curves mean +/− standard deviation. The gray shaded area in (Figure 4g_7,_g_8_,h_7_,h_8_), represent the CO_2_ on time period. The scale bars in (Figure 4b to Figure 4f) represent 2 mm, and the scale bars in (Figure 4g,h) represent 0.5 mm. CBV: cerebral blood volume; rCBV: relative cerebral blood volume; CBF: cerebral blood flow; ULM: ultrasound localization microscopy; ROI: region‐of‐interest; SO_2_: oxygen saturation; rSO_2_: relative oxygen saturation; MSPA: multispectral photoacoustic; CO_2_: carbon dioxide.

With the animal under anesthesia (1–1.5% isoflurane mixture with O_2_), we conducted a craniotomy procedure on all four animals and acquired continuous interleaved UFUS and MSPA imaging during the 8‐min CO_2_ stimulation paradigm (2 min of baseline, 3 min of 5% CO_2_ stimulation, and 3 min of post‐stimulation, see Experimental Section). An example result from one animal is shown in **Figure** **4**, which was acquired at around Bregma −5.8 (Figure 4a). Following the hypercapnia stimulation, using intravenous MB injection, ULM imaging was obtained to map microvascular anatomy and vertical flow directions (Figure 4b). After data acquisition, UFUS data was processed to provide both CBV and CBF maps as shown in Figure 4c,d, respectively, and the MSPA data was spectral unmixed to provide SO_2_ maps (Figure 4d) of the brain during the stimulation. In (Figure 4b) ULM and (Figure 4e) CBF images, the red and blue colors denote downward and upward flows, and in the cortical regions these colors represent arterioles and venules, respectively.

We further calculated the relative changes in CBV, CBF, and SO_2_ maps—referred to as rCBV, rCBF, and rSO_2_, respectively as proxies for CVR measurement (Experimental Section).^[^ ^28^ ^]^ Although the stimulation increased the CBV, CBF, and cerebral SO_2_ globally (Videos S1–S3, Supporting Information), differential temporal CVR changes were observed between singular cortical vessels, as shown in the representative rCBV map. To further study the regional differences in the CVR using obtained multiparametric information, we analyzed CBV, CBF, and SO_2_ individually in the ROI 1 and ROI 2 (green and orange boxes) as shown in Figure 4g,h. During the hypercapnia stimulation, the rCBV showed ≈100% increase in ROI 1 (Figure 4g_4,_g_7_) and ≈120% increase in ROI 2 (Figure 4g_4_,g_7_), and overall rSO_2_ synergistically increased ≈80% for ROI 1 (Figure 4g_5,_g_7_) and ≈60% for ROI 2 (Figure 4g_5_) and (Figure 4g7). Furthermore, we delineated ROIs around the singular vein and artery in ROI 1 (Figure S9a,b, Supporting Information) based on a high‐resolution ULM microvascular map (Figure 4g_1_) and observed that rCBV changes in the vein (≈15% increase) were much less compared to the artery (≈160% increase) at the peak stimulation time point (*t* ≈260 s). In contrast to the CBV changes, the analysis of CBF (Figure 4g_2,_g_3_,g_6_,h_2,_h_3,_h_6_); Figure S9d–f, Supporting Information) showed different dynamics, where the arterial CBF demonstrated lower relative increase (≈31.6% increase for ROI 1, from ≈9.5 to ≈12.5 mm s^−1^—(Figure 4g8), and ≈25.6% increase for ROI 2, from ≈9 to ≈11.3 mm s^−1^—(Figure 4h8) comparing to that of the veins (≈91.7% increase for ROI 1, from ≈6 to ≈11.5 mm s^−1^—(Figure 4g8), and ≈122.2% increase for ROI 2, from ≈4.5 to ≈10.0 mm s^−1^—(Figure 4h8)). These differential CBF changes also held valid when drawing ROIs around a singular arteriole and venule (Figure S9d–f, Supporting Information).

Surprisingly, we observed strong reductions of rCBV during hypercapnia in the superior sagittal sinus (SSS) (Figure S10, Supporting Information) and the ventricle (Figure S11, Supporting Information) regions of the brain. Although both SSS and ventricle regions exhibited strong CBV intensity (Figures S10a and S11a, Supporting Information), the rCBV calculated during hypercapnia showed a decrease of ≈−40% for SSS (Figure S10e,g, Supporting Information) and ≈−17% for ventricle (Figure S11e,g, Supporting Information) regions. On the other hand, the rSO_2_ for SSS showed an increase of ≈50% (Figure S10f,g, Supporting Information), but minimal to no change was shown for the ventricle region (Figure S11f,g, Supporting Information). This is likely because SSS consists of large veins that drain a significant amount of blood, whereas the ventricle region primarily consists of the complex capillary bed (see enlarged ULM image of the ventricle in Figure S11d (Supporting Information) in the choroid plexus that produces cerebrospinal fluid (CSF), which does not absorb sufficient light to generate photoacoustic contrast.

### 2.5 Correlation Between Cortex and Vein Temporal Dynamics During the Resting and Stimulation States

**Figure 5:**
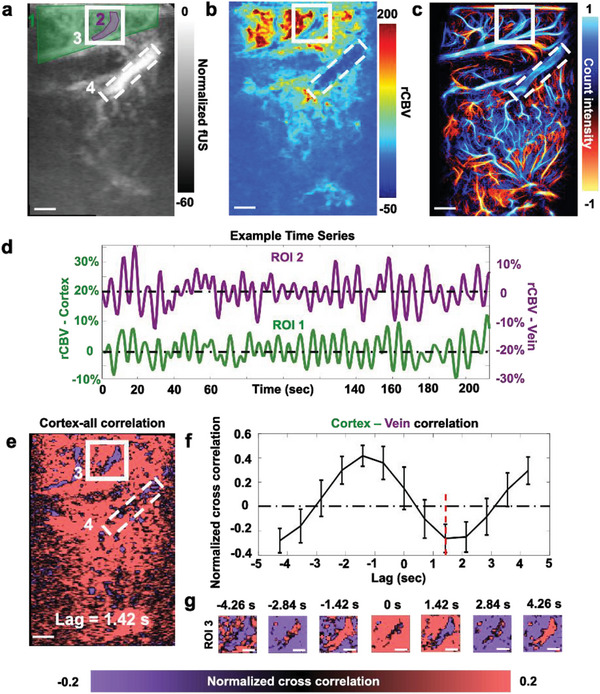
Correlation between the venous and cortical hemodynamics revealed by fUSPA. Data in this figure are acquired with the fSUPA device positioned to image sagittal section of the brain at around the midline. a) The CBV map. The cortical CBV dynamics were averaged across the green‐shaded area (ROI 1) and the venous CBV dynamics were extracted from the purple‐shaded area (ROI 2) within the white solid ROI (ROI 3). The ventricle region is delineated with a white dashed ROI (ROI 4). b) The rCBV change map during the hypercapnia stimulation. c) The corresponding ULM image showed the vertical flow direction in the sagittal plane. d) The time activity of the averaged cortical rCBV (in green, from ROI 1) and averaged venous rCBV (in purple, from ROI 2). e) The cross‐correlation map between the temporal dynamics of the averaged cortical CBV and the temporal dynamics of each voxel in the CBV image, with the strongest coupling at Lag = 1.42 sec is displayed in (e). f) The cross‐correlation plot between the cortex (ROI 1) and the venous (ROI 2) CBV signals. The strongest decorrelation was highlighted with the red dashed line at Lag = 1.42 s. g) The enlarged cross‐correlation map of ROI 3 at different lags. The scale bars in Figure 5a–c) and (e) represent 1 mm, and the scale bars in (g) represent 0.5 mm. ROI: region‐of‐interest; CBV: cerebral blood volume.

The fUSPA system was further employed to study the hemodynamic changes in the sagittal section near the midline of the rat brain. The CBV map and the corresponding rCBV map during hypercapnia stimulation are shown in **Figure** **5**a,b, respectively, and the ULM‐based vertical flow map is shown in Figure 5c. Similar to the coronal plane imaging results, the overall cortex (green shaded area – ROI 1 in Figure 5a) showed an increase in rCBV, while the veins (example vein shown as purple shaded ROI 2 area within the white solid ROI 3 box of Figure 5a–c exhibited minimal rCBV change (Figure 5b). The veins in the ROI 2 were identified using the ULM vertical flow map (Figure 5c), and corresponding lower SO_2_ value is shown in Figure S12 (Supporting Information). Similar to the previous observation in Figure 4, although the ventricle region (white dashed inlet—ROI 4 in Figure 5a–c showed strong CBV intensity (Figure 5a), negative rCBV and little SO_2_ content (Figure S12, Supporting Information) were found during hypercapnia stimulation (Figure 5b).

fUSPA also allowed us to study the correlation between the veins (ROI 2) and overall cortical hemodynamics (ROI 1) during the resting state by performing continuous UFUS data acquisition for 5 min on the anesthetized (1–1.5% isoflurane) animal without stimulation. In the rCBV time series maps derived from continuous UFUS data, the averaged rCBV intensity for cortical (ROI 1) and venous (ROI 2) regions plotted as a function of time are plotted in Figure 5d and demonstrated a clear out‐of‐phase dynamics (Figure 5d). To further analyze this temporal pattern across the imaging plane, we calculated the cross‐correlation between the averaged cortical (ROI 1) dynamics and all voxels in the CBV map, denoted as cortex‐all correlation (full lag maps in Figure S13, Supporting Information), example Lag = 1.42 s in Figure 5e, Experimental Section) and the cross‐correlation between the cortical and venous dynamics, denoted as cortex‐vein correlation (Figure 5f, Experimental Section). Interestingly, although the majority of the voxels showed positive correlations with the cortical regions (Figure 5e; Figure S13, Supporting Information), the venous dynamics, however, were clearly anti‐correlated with other regions at all Lags (Figure 5e,g, Figure S13, Supporting Information), with cortex‐vein correlation showed a positive peak at Lag = −1.42 s (*R* = 0.37, (Figure 5f)) and a negative peak at Lag = +1.42 s (*R* = −0.26, red dashed line in Figure 5f). This lag observed in the temporal pattern aligns with previous measurements of transit time in rodents.^[^ ^32^ ^]^ However, the slow wave cortex‐vein coupling, as obtained using high spatiotemporal resolution fUSPA, distinguishes itself from other imaging modalities.

## 3 Discussion

In this article, we present a novel in vivo multimodal fUSPA imaging system to quantitatively map multiparametric hemodynamic and molecular information of deep brain regions of rats with high spatiotemporal resolutions. Effective integration of hardware and software components of fUSPA enabled interleaved UFUS and MSPA data acquisitions in real‐time using a single head‐mountable imaging device. UFUS data processing provided both power Doppler‐based CBV mapping and ultrasound velocimetry‐based CBF mapping at single vessel resolution. The spectral unmixing of MSPA data provided cerebral SO_2_ maps. Furthermore, through the intravenous administration of FDA‐approved ultrasound and optical contrast agents, MBs, and ICG, respectively, fUSPA system allowed ULM‐based super‐resolved microvasculature imaging and ICG molecule‐enhanced vascular perfusion imaging of the brain. In total, the fUSPA imaging system can provide the following six brain maps: CBV, CBF, SO_2_, ICG vascular perfusion, super‐resolved microvascular anatomy, and axial blood velocity. Such comprehensive mapping of individual and dissected hemodynamic parameters provides fUSPA imaging distinct advantages over the BOLD fMRI, which represents a mixed effect from CBV, CBF, and cerebral metabolic rate of oxygen consumption (CMRO_2_) that confound interpretations.^[^ ^5^, ^6^, ^33^ ^]^

Using high‐resolution multiparametric hemodynamic imaging capabilities of the fUSPA system, we investigated brain‐wide CVR differences elicited by hypercapnia stimulation. Traditionally, fMRI,^[^ ^34^ ^]^ optical imaging,^[^ ^35^ ^]^ or a combination of these modalities^[^ ^36^ ^]^ are used to study the hypercapnia effect on different brain regions or global cerebrovascular reactivity. However, using fUSPA, which offers superior imaging depth compared to pure optical imaging and improved spatiotemporal resolution and high sensitivity compared to fMRI, we could identify individual vessel‐specific effects of hypercapnia that other modalities often miss. As hypercapnia is a known vasodilator and increases tissue oxygenation,^[^ ^31^ ^]^ our results confirmed a global and synergistic increase in CBV, CBF, and SO_2_ (Videos S1–S3, Supporting Information), but revealed regional differences—e.g., the SSS region exhibited a decrease in rCBV but increase in rSO_2_ values during the hypercapnia stimulation. Further leveraging the high‐resolution of the fUSPA system, single vessel analyses were conducted and differential hemodynamic response during stimulation was found between the cortical arteries and veins. Strikingly, cortical veins exhibited minimal rCBV increase but significant rCBF increase compared to the arteries, and these observations were replicable across all *n =* 4 experimental animals. The revealed rCBV differences between arteries and veins were similar to other sensory stimulation,^[^ ^37^ ^]^ and the observed differential CBF and CBV changes agreed with previous hypercapnia studies based on Doppler ultrasound^[^ ^38^ ^]^ and positron emission tomography,^[^ ^39^ ^]^ respectively. However, our results show the first evidence of multiparametric hemodynamic changes at a single vessel spatial resolution, making it suitable to study microvascular cerebral metabolisms^[^ ^40^ ^]^ and brain injuries.^[^ ^41^ ^]^

The advantage of deep brain single vessel multiparametric information further extended to imaging the resting state brain in the sagittal plane. The analysis of continuous UFUS data during the rat resting state showed the venous dynamics were out‐of‐phase from the overall cortical dynamics with ≈1.42 s delay. Since CBV is directly proportional to the volume changes of the moving scatterers (where hemoglobins are the main scatterers in the vessels), this consistent lag between venous (ROI 2 in Figure 5) and cortical dynamics (ROI 1 in Figure 5) suggested a reproducible temporal pattern between the venous blood volume and overall CBV, which further agreed with previous findings using optical imaging^[^ ^32^ ^]^ and fMRI.^[^ ^3^ ^]^ However, optical imaging is not capable of mapping deep brain venous dynamics, while fMRI could not directly assess the venous rCBV change,^[^ ^3^ ^]^ setting fUSPA as a unique device for studying the temporal patterns of the single vessel in the deep brain, which is often assessed in sleep,^[^ ^42^ ^]^ and resting state studies.^[^ ^43^ ^]^ Furthermore, fUSPA provided the multiparametric ULM‐based vertical speed map and MSPA‐based SO_2_ map that helped us confidently segment out the venous area, which was not achievable with fMRI alone.

Remarkably, both ventricle and venous regions showed decreased rCBV during hypercapnia stimulation (Figures 4 and 5b; Figure S11b, Supporting Information) and decorrelation with the cortical dynamics during the resting state (Figure S13, Supporting Information). While the CBV intensity was strong in the whole ventricular region (Figure S11a, Supporting Information; Figure 5a), the correlation maps (Figure 5e–g) and the SO_2_ map (Figures S11 and S12, Supporting Information) showed only low and scattered values. Considering the web‐like microvascular structures in the ventricle mapped by ULM, we hypothesize that the strong CBV signal was primarily from the fast‐flowing blood in the choroid plexus capillaries that resided in the ventricle.^[^ ^44^ ^]^ Many prior studies reported that the venous dynamics were coupled and correlated with the CSF signal,^[^ ^45^ ^]^ especially during deep/forced breathing. Therefore, given that the choroid plexus is responsible for the production of the majority of CSF,^[^ ^46^ ^]^ we reasonably hypothesize that the venous‐ventricle correlation measured by CBV mapping was related during isoflurane anesthesia. However, this hypothesis warrants further studies.

The fUSPA imaging system reported here does have some limitations. First, although our light delivery is compactly integrated into the transducer device, it still requires a few millimeters of coupling medium (working distance) for proper illumination. This distance not only hinders the system's ability for mobile animal imaging but also increases the data transfer size, leading to a reduction in temporal resolution. To address this, future improvements can employ a transparent ultrasound transducer (TUT) array,^[^ ^47^ ^]^ which would allow seamless integration of multimodal UFUS and MSPA with minimal coupling distance. Furthermore, a multi‐angle light illumination through the TUT array can help improve the depth of MSPA imaging. Second, the vascular compartment segmentation, specifically distinguishing between veins and arteries, is applicable only for cortical vessels since the ULM and CBF map would only be able to encode the vertical flow direction (upwards/ascending or downwards/descending) relative to the cortical surface. This limitation may be mitigated by generating 3D ULM images of the entire brain using a 2D matrix probe,^[^ ^48^ ^]^ and then encoding 3D flow direction to further understand the blood‐flowing pathway.^[^ ^49^ ^]^ Third, due to the need for averaging five sets of multispectral PA frames to increase the SNR, the temporal resolution of the combined fUSPA system is 3.3 s per frame. Although this temporal resolution is sufficient for assessing CVR induced by CO_2_ stimulation, it is not capable of mapping transient neural activity changes, such as epilepsy.^[^ ^9^ ^]^ The MSPA imaging temporal resolution can be increased using a high pulse repetition (30 Hz) nanosecond pulsed OPO laser. Lastly, the resolution of the fUSPA system can be further improved by incorporating high‐frequency (>25 MHz) ultrasound transducers to help map finer vessels.^[^ ^50^ ^]^

Despite the above‐mentioned limitations, our multimodal fUSPA system is the first of its kind that enables deep brain comprehensive hemodynamic mapping and exogenous contrast‐enhanced brain imaging with high resolution and sensitivity. The unique multiparametric capabilities of fUSPA will help advance our understanding of complex brain functions, thereby paving the way for better insights into various neuro diseases, such as brain cancer, sleep disorders, and Alzheimer's disease.

## 4 Experimental Section

### fUSPA Imaging Device

To achieve a compact fUSPA imaging head, a light‐weight portable holder was designed that accommodated ultrasound transducer (UST) in the center and fiber optic light delivery through acrylic lenses from either side of the UST to focus light into the ultrasound detection zone (Figure S1a,b, Supporting Information). The high‐frequency UST (Vermon, Tours, France, 15 MHz, 40% fractional bandwidth, 128 elements with 0.1 mm pitch, 1.5 mm elevation, and 6 mm elevational focus) was custom designed, and the overall size of UST was 5 mm × 20 mm × 25.4 mm (width × length × height) with a weight of ≈5 grams. The high‐frequency UST was used for both UFUS plane wave imaging and PA detection. The fiber optic delivery consisted of four rectangular fiber bundles (Fiberoptic Systems Inc, Simi Valley, CA, USA), each with a size of 2 mm × 5 mm, cased inside a 2.5 mm × 6 mm stainless steel tubing, and inserted into the holder on each side (Figure S1a,b, Supporting Information). Two 50‐degree lenses machined from polished acrylic sheets were attached to the front of the optical fiber bundles to refract the optical beams such that they focus at ≈6–7 mm away from the transducer surface, and allow co‐alignment with the ultrasound field inside the tissue medium (Figure S1c,d and Section S1, Supporting Information for the calculations). The focused beam at ≈7 mm was measured to cover an area of ≈3 × 26 mm^2^, which was similar to the theoretical calculation of 2.1 × 20 mm^2^. For animal imaging, the laser energy was controlled by tuning the Q‐switch delay, and the output laser energy after the lenses were measured by an energy meter (PE50BF‐DFH‐C, Ophir‐Spiricon, LLC, North Logan, UT, USA) to be less than 15 mJ pulse^−1^, which gave an optical fluence below the American National Standards Institute (ANSI) safety maximum permissible exposure (MPE) limit of 20 mJ cm^−2^.^[^ ^51^ ^]^

### Interleaved UFUS and MSPA Data Acquisition

A Vantage 256 ultrasonic research system (Verasonics Inc., Kirkland, WA, USA) was custom programmed to perform interleaved MSPA and UFUS imaging, including transmitting ultrafast plane wave angle compounded US imaging, receiving US echoes and PA pressure waves, and online–offline reconstruction using pixel‐based beamforming. The schematic of the combined fUSPA imaging sequence for interleaved MSPA and UFUS data acquisition is shown in Figure S2 (Supporting Information) and described in detail below.

Each fUSPA frame data was acquired in 3.3 s and consisted of 1.9 s for MSPA data, ≈1.2 s for UFUS data acquisition, and ≈0.2 s for trigger synchronization (Figure S2a, Supporting Information). As shown in Figure S2b (Supporting Information), each MSPA data block was composed of 16 dual‐modality B‐mode US and PA (USPA) frames, with each B‐mode US and B‐mode PA frame acquired every 100 ms (Figure S2b, Supporting Information), limited by the 10 Hz pulse repetition rate of the tunable optical parametric oscillator (OPO) laser (Phocus Mobile, Opotek Inc., Carlsbad, CA, USA; 5–7 ns pulse length, peak pulse energy of 100 mJ at 750 nm, with tuning range of 690 to 950 and 1200 to 2600 nm). While B‐mode US frame shows mechanical impedance contrast based on anatomical information of the tissue, B‐mode PA frame displays optical absorption contrast based on molecular information of the tissue. Since both B‐mode US and PA frames were acquired using the same UST probe, a co‐registered US+PA (USPA) frame with overlaid anatomical and optical contrasts was helpful in understanding the origin of PA signals.^[^ ^16^, ^47^ ^]^ After acquiring each USPA frame data, the Vantage was set to idle to wait for the laser trigger pulse (Figure S2b, Supporting Information), which was achieved by using an external function generator (SDG6022X, Siglent Technologies NA, Solon, OH, USA) as the master (similar to the Verasonics recommended strategy^[^ ^52^ ^]^) to minimize jitters for PA signal acquisition at other wavelengths. The fast‐tuning feature of the OPO laser allowed us to tune the wavelength for every 100 ms, enabling real‐time MSPA imaging. Three wavelengths—750, 800, and 850 nm—were chosen for MSPA imaging, and 15 MSPA frames (5 sets of 750, 800, and 850 nm PA frames) were acquired to achieve a sufficient signal‐to‐noise ratio. One additional MSPA frame was acquired due to potential frame loss during laser trigger synchronization, giving a total of 16 MSPA frames. With the 10 Hz laser pulse repetition frequency, 16 MSPA frames (along with respective US frames) took 1.6 s, and 0.3 s were taken to transfer and save the data, which totaled up to ≈1.9 s shown in Figure S2b, Supporting Information.

Then UFUS data was acquired right after the MSPA data transfer. The timing diagram of the UFUS data acquisition sequence block is displayed in Figure S2c (Supporting Information). During one UFUS acquisition, four half cycles of 11 volts were used to excite the ultrasound transducer^[^ ^53^ ^]^ with seven planar ultrasonic waves tilted at angles varying from −6 to 6° (−6° −4°, −2°, 0°, 2°, 4°, and 6°, with 75 µs between each plane wave angle, which were coherently summed to generate one compounded US frame every 525 µs. 150 such compounded frames (US_C1_–USC_150_ as shown in Figure S2c, Supporting Information) were used to generate one power Doppler‐based CBV map in 78.75 ms^[^ ^9^ ^]^ and an additional 1.1 s was required for data transfer (Figure S2c, Supporting Information). Then the system was set to idle and awaited the trigger for synchronization and ready for the next fUSPA frame acquisition.

### MSPA‐based Molecular Imaging

Linear unmixing based on non‐negative least squares curve fitting was used to unmix different molecular distributions (HbO, HbD, and ICG) inside the tissue. The input matrix for curve fitting was initially obtained from omlc.org and normalized based on the measured OPO laser pulse energy differences across the three wavelengths (750, 800, and 850 nm). As discussed in the above section and (Figure S2b, Supporting Information), each MSPA data block of 1.6 s duration consisted of 15 PA images, acquired in 5 sets of 3 PA images (one PA image per each of the three wavelengths). The resulting 5 PA images for each of the three wavelengths were averaged to improve the signal‐to‐noise (SNR), and averaged three‐wavelength PA images were used in the spectral unmixing to generate unmixed images of HbO, HbD, and ICG. While the unmixed ICG map was directly displayed after log compression (e.g., Figure 3d), the SO_2_ map was generated from the unmixed HbD and HbO images, by calculating the SO_2_ = HbO/(HbO + HbD) and normalization across all the SO_2_ values for each pixel (e.g., Figure 2d).

### UFUS‐based CBV Imaging

For each UFUS data block, one power Doppler map was generated as a CBV proxy using similar processing as the existing literature. We coherently compounded the in‐phase/quadrature (IQ) data and then applied a singular value decomposition (SVD) filter^[^ ^54^ ^]^ to remove the tissue motion (first nine ranks) from the 150 compounded frames while preserving the signal components associated with the moving blood signal. As red blood cells were the main scatterers in the blood, power Doppler signal intensity was considered to be directly proportional to the fractional moving CBV.^[^ ^9^ ^]^ Hence, the power Doppler intensity map was considered as the CBV intensity map.

### UFUS‐based CBF Imaging

To study the dynamic CBF changes, a velocimetry map was derived from the acquired UFUS data. The processing was similar to the previously reported method by Tang et al.^[^ ^12^ ^]^ In summary, the algorithm fits autocorrelation functions using nonlinear regression to both the positive and negative components of the IQ temporal signal from the 150 compounded frames to solve the *z*‐direction velocity.

In detail, each UFUS compounded frame data was spatiotemporally filtered using SVD by rejecting components corresponding to the first ten singular values, followed by a fourth‐order Butterworth 70 Hz high pass filter to generate the moving blood IQ signal. Then the IQ signal was subjected to a directional filter to obtain positive and negative signal components based on the frequency spectrum, as a clear separation of the opposing frequency components was observed based on the blood flow direction relative to the UST.^[^ ^9^ ^]^ The positive and negative signals were only considered valid for later processing if their individual positive and negative frequency powers were greater than 0.2 and 0.25 of the total frequency power, respectively. In addition, to remove the effect of noise, only the signals with the first temporal autocorrelation >0.2 were used in subsequent processing.^[^ ^12^ ^]^ Then a first‐order temporal autocorrelation function was used to separately fit the filtered signals to solve the *z*‐direction velocity as velocity maps as well as their corresponding flow direction information. The velocity maps generated by this fitting procedure were found to agree with the vertical speed obtained from the ULM maps.^[^ ^12^ ^]^

### ULM Imaging

ULM was acquired using the same fUSPA system with the help of intravenous injection of ultrasound contrast agents – MBs into rats. The ULM data acquisition was similar to the above‐described UFUS data acquisition, except with minor changes in the time between the angles for plane wave imaging and the number of compounded frames. In particular, ULM imaging was performed using plane waves transmitted at 7 angles (−6° −4°, −2°, 0°, 2°, 4°, and 6°) with 200 µs between each angle to generate compounded images with a frame rate of ≈700 Hz. 400 compounded images (560 ms duration) were acquired for each sequence and then ≈2 s were used to transfer the data to the host, which gave a sequence duration of ≈2.6 s. 300 such sequences were acquired, thereby giving a total ULM accumulation time of 168 s with a total acquisition time of ≈9 min. The ULM images were obtained from beamforming of the raw RF data using the Verasonics system to generate the IQ data. The IQ dataset was filtered using the spatiotemporal SVD filter,^[^ ^54^ ^]^ where the first few singular values were removed to isolate MB signals from the surrounding tissue signals. ULM MB localization procedure was similar to.^[^ ^24^, ^55^ ^]^ In brief, MBs were localized to sub‐pixel resolution using spline‐based interpolation. Thereafter, the Hungarian algorithm was used to form tracks^[^ ^56^ ^]^ with a minimum duration of 100 s. The tracks were then accumulated to form the vasculature map.^[^ ^55^ ^]^ The displacement of MBs between two consecutive frames was used to estimate the speed of tracked MBs, and the speed of all MB traces during the ULM session was averaged to form the flow speed map.^[^ ^57^ ^]^

### Resolution Wire Targets

The resolution of the US and PA was characterized by imaging 50 µm diameter micro metal wire targets embedded at different depths inside a tissue‐mimicking phantom (Figure S4, Supporting Information). To generate strong ultrasound and photoacoustic contrasts, three 50 µm diameter micro metal wires (W1–W3 as shown in Figure S4a, Supporting Information) were dyed with India ink and then positioned ≈5 and 4 mm apart from each other on the axial and lateral planes, respectively. Then a solution mixture of agar, intralipid, and silica beads with a respective weight ratio of 1.5%, 1%, and 1% was poured into the tank to provide the ultrasound background speckles as well as the optical scattering.^[^ ^47^ ^]^ While the ultrasound imaging contrast of the phantom was based on the acoustic impedance differences between the wire and the background, the photoacoustic imaging contrast was based on the light absorption of metal wire targets dyed with India ink.

### Field‐of‐View Targets

The FOV of PA imaging was characterized by imaging India ink dyed microwire targets placed orthogonally to the imaging plane of an area of 20 mm wide by 25 mm deep. Micrometal wires were spaced 5 and 3 mm between each other in the axial and lateral planes, respectively, to minimize interference of PA signal while maintaining the sufficient density of the targets. Then the FOV characterization was performed in an optical scattering medium made of deionized water with 1% intralipid. PA imaging was conducted at 750 nm wavelength.

### Tube Phantom of Flowing Blood and Static ICG

To validate the functional and molecular imaging capabilities of UFUS and MSPA modalities of the fUSPA system, a tube phantom consisting of two polyethylene tubes (outer diameter: 2.08 mm, inner diameter: 1.57 mm, PE 205, BD Medical, Franklin Lakes, NJ, USA) was developed. One tube (left side in Figure S5a, Supporting Information) was filled with defibrinated bovine blood (Lampire Biological Laboratories, Pipersville, PA, USA) while circulated using a peristatic pump (model 3386, Cole‐Parmer, Vernon Hills, IL, USA). The other tube (right side in Figure S5a, Supporting Information) was filled with 1290 µm ICG in deionized water. The tubes were embedded inside a solid phantom, made of 1.5% agar and 1% intralipid to mimic optical scattering, to reduce any motion artifacts caused by the pump. To induce the blood oxygenation differences, a gas chamber was added to the blood circulation pathway in the left tube. The gas chamber was filled with a mixture of CO_2_ and O_2_ with their respective ratio controlled by individual flow meters. The output flow rate was maintained constant at 3 L min^−1^. For example, 100% O_2_ was achieved by giving O_2_ at a flow rate of 3 L min^−1^, while a mixture of 66% O_2_ and 33% CO2was achieved by giving O_2_ at 2 L min^−1^ and CO_2_ at 1 L min^−1^. As previously described in methods 4.2, the interleaved MSPA (with 750, 800, and 850 nm) and UFUS data acquisition of the tube phantom was performed using the fUSPA imaging system. While the MSPA data of each gas mixture concentration was processed with the methods described in 4.3 to provide unmixed maps of HbO, HbD, SO_2_, and ICG molecular distribution, the UFUS data was processed with methods described in 4.4 to generate the blood flow map.

### Animal Handling and Surgery

All animal procedures were approved by the Pennsylvania State University Institutional Animal Care and Use Committee (IACUC), IACUC protocol number: PROTO201800178. Adult Sprague Dawley rats (*n =* 4, 10–16 weeks old) were used in the experiments and acclimated for at least 5 days prior to the surgery. These animals received craniotomy survival surgery prior to imaging sessions to allow US and PA imaging of the brain. On the date of the surgery, the animal was anesthetized by administering isoflurane/O_2_ via vaporizer at 3–5% and maintained at 1–2% throughout the surgery.

A sagittal skin incision was performed across the posterior part of the head to expose the skull, and a dental drill was used to mark the window for craniotomy with a size up to 10 mm × 14 mm. Beginning with a 1.0 mm drill bit, a rectangular cut was made within the marked borders of the craniotomy. The drilling was frequently paused to cool the skull bone with cold saline to reduce heat damage, edema, and to control bleeding around or underneath the skull. The thinning was continued until the pial vessels were clearly seen through the thinned skull, the bone flap was lifted off using fine tip forceps and exposed the underlying intact dura. Then 1.5% weight ratio agar (UltraPure Agarose, Invitrogen, Waltham, MA, USA), mixed in sterilized saline was molded into a cuboid with a similar thickness to the skull and cut into a size slightly smaller than the window. The agar cuboid was then placed on top of the dura to provide hydraulic pressure and acoustic coupling. Then the whole window on the animal head was covered by a 50 µm thick PMP film (Goodfellow, Pittsburgh, PA, USA) as a skull prosthetic and acoustic transparent window. Next, the acoustic window was sealed to the surrounding skull using dental cement (C&B metabond, Parkell Inc., Brentwood, NY, USA). A nut was placed at the Bregma as an anatomical marker for in vivo imaging sessions. Following the surgery, the animal was allowed one week to recover before the start of any imaging experiments.

### Animal Imaging and CO_2_ Stimulation

At the beginning of the imaging session, the animal was anesthetized using 3% isoflurane mixed with medical oxygen at 2 L min^−1^. Once the animal was stably anesthetized, it was moved to the imaging stage and fixed to the stereotaxic frame with a nose cone providing a continuous supply of 1–1.5% isoflurane mixed with medical oxygen at 2 L min^−1^ to maintain anesthesia throughout the imaging session. Then the imaging window was gently cleaned with sterilized saline using cotton‐tipped applicators. Warm ultrasound coupling gel mixed with saline was then applied over the window to couple the fUSPA probe to conduct UFUS, MSPA, and ULM imaging. During the UFUS and MSPA imaging sessions, the hypercapnia (CO_2_) was performed by delivering 5% CO_2_ mixed with oxygen through the nose cone. The 5% CO_2_ volume fraction was controlled by adjusting the flow rate of the O_2_ and CO_2_ using individual flow meters. UFUS and MSPA imaging data was continuously acquired during an 8‐min stimulation paradigm, which was composed of a 2‐min baseline, a 3‐min CO_2_ stimulation period, and a 3‐min post‐stimulation period.

For ULM imaging, a 26‐gage intravenous (IV) catheter needle was placed in the rat tail vein for MB injection. MBs (SIMB 4–5, Advanced microbubbles, Newark, CA, USA) were diluted to 10^8^ MBs mL^−1^ following the manufacturer's protocol. Two bolus injections of 400 µL MBs were performed at *t* = 0 (at the start of ULM imaging) and *t* = 5 min, respectively. Then, as described above, the ULM imaging sequence was performed to obtain super‐resolved microvasculature images of the brain.

To investigate the exogenous photoacoustic molecular imaging capabilities of the fUSPA imaging system, the Food and Drug Administration (FDA) approved optical fluorescence contrast agent, ICG, was intravenously administered while the animal was maintained under isoflurane anesthesia. The 10 min MSPA imaging protocol was performed to delineate the depth‐dependent intrinsic (HbO and HbD) and exogenous (ICG) molecular contrast information. After a 1‐min baseline acquisition of MSPA imaging data, 500 µL of 129 µm ICG in sterilized saline was slowly injected through the catheterized tail vein (≈16.7 µL s^−1^). Following the completion of the injection, MSPA imaging sequence was continuously performed for 8:30 min to study post‐injection time activity of intravascular ICG inside the brain.

### Ex Vivo Isolated Rat Brain Imaging

We performed ex vivo whole rat brain imaging experiments to help understand and optimize fUSPA device imaging capabilities for in vivo rat brain imaging. The rat brain was carefully removed from the head following CO_2_ euthanasia according to the approved IACUC protocol. Immediately after the removal, the whole brain was embedded inside a 1.5% agar medium to provide additional stability and easy coupling to the fUSPA device. Next, dual‐modality US and MSPA imaging of the ex vivo brain was performed using the fUSPA system.

### Cross‐Correlation Function for Cortex‐Vein Temporal Dynamics

To investigate the coupling between the temporal dynamics of the veins and the overall cortical vessels, continuous UFUS imaging was performed of the rat brain at high temporal resolution (≈0.71 s per frame). The animal was maintained under anesthesia using 1–1.5% isoflurane while the continuous UFUS imaging was performed for 5 min without any stimulation. The acquired data were then analyzed offline to investigate the correlation between the venous and cortical vessel CBV activities. As shown in Figure 5, four ROIs were marked for further analysis. The ROI surrounding the cortex was denoted as ROI 1, and ROI surrounding a single vein (as confirmed with ULM, Figure 5c) was denoted as ROI 2. ROI 3 in the cortex encompasses ROI 2 and was used to help visualize the anti‐correlation effect from the vein shown in ROI 2 (Figure 5g). ROI 4 marks the ventricle region. The temporal dynamics of the cortex and vein were obtained by averaging the time activities of the ROI 1 and ROI 2 voxels, respectively. The example averaged time series are shown in Figure 5d. The cross‐correlation function was done for two scenarios: 1) cortex‐all correlation, where the temporal dynamics of each individual voxel was cross‐correlated with the time‐averaged cortical dynamics (from ROI 1), and the resulted correlation coefficient (*R*) was plotted as the map for different lags (Figure 5e; Figure S13, Supporting Information). 2) cortex‐vein correlation, where the cross‐correlation was calculated between the averaged cortical vascular dynamics from ROI 1 and the averaged venous dynamics from ROI 2 (Figure 5f,g).

### Statistical and Data Analysis

The saved UFUS and MSPA data were processed offline to extract the time dynamics of the rCBV, rSO_2_ (or relative ICG changes, e.g. Figure 3e), and CBF changes. While UFUS data were processed to obtain the CBV and CBF maps of each time point, MSPA data were processed to obtain the SO_2_ map of each time point. Relative change in the intensity of CBV maps of each pixel was calculated by:

(1) $$rCBV\;=\left(CBV\left(t\right)-\frac{\sum_{t=1}^{10}CBV\left(t\right)}{10}\right)\;/\frac{\sum_{t\;=\;1}^{10}CBV\left(t\right)}{10}\times 100\%$$

where CBV(*t*) denotes the power Doppler intensity or CBV intensity of the pixel at time *t* obtained from the power Doppler intensity. Similarly, rCBF and rSO_2_ were calculated by:

(2) $$rCBF\;=\left(CBF\left(t\right)-\frac{\sum_{t=1}^{10}CBF\left(t\right)}{10}\right)\;/\frac{\sum_{t\;=\;1}^{10}CBF\left(t\right)}{10}\times 100\%$$

and

(3) $$r\mathrm{SO}2\;=\left(\mathrm{SO}2\left(t\right)-\frac{\sum_{t=1}^{10}\mathrm{SO}2\left(t\right)}{10}\right)\;/\frac{\sum_{t\;=\;1}^{10}\mathrm{SO}2\left(t\right)}{10}\times 100\%$$

respectively for each pixel. Then all the relative changes of the pixels in ROIs were averaged and shown as solid curves in Figure 4, and the mean ± standard deviation of these pixel‐based relative changes are shown as shaded areas. Data were acquired and analyzed on a sample size of *n =* 4 animals, representing the replicability and reproducibility. All the analyses were done using MATLAB R2022b (Mathworks, Natick, MA, USA).

## Conflict of Interest

The authors declare no conflict of interest.

## Author Contributions

The conceptualization of the project was undertaken by H.C. and S.R.K., the software and data analysis were carried out by H.C., S.M., P.G., S.A., X.L., and T.X. Investigation tasks were managed by X.L., P.J.D., N.Z., B.J.G., and S. R.K. Data curation was handled by H.C., S.M., and V.N. The original‐draft preparation was completed by H.C. and S.M. Experiment design was executed by H.C., Q.L., and S.R.K., with hardware managed by H.C., M.L., J.L., B.J.G., and W.T. S.R.K. provided supervision throughout the project, also overseeing project administration. Funding acquisition was accomplished by B.J.G. and S.R.K.

## Supporting information

Supporting Information

Supplemental Video 1

Supplemental Video 2

Supplemental Video 3

## Data Availability

Data underlying the results presented in this paper are publicly available at the Github repository: https://github.com/pramethg/FUSPA.git.
